# Prognostic value of pretreatment ^18^F-FDG PET/CT metabolic parameters in esophageal high-grade neuroendocrine carcinoma: A bicenter retrospective study

**DOI:** 10.3389/fonc.2023.1145557

**Published:** 2023-03-13

**Authors:** Guozhu Hou, Ningning Zhao, Fang Li, Hongli Jing, Rong Zheng

**Affiliations:** ^1^ Nuclear Medicine Department (PET-CT Center), National Cancer Center/National Clinical Research Center for Cancer/Cancer Hospital, Chinese Academy of Medical Sciences and Peking Union Medical College, Beijing, China; ^2^ Nuclear Medicine Department (PET-CT Center), National Cancer Center/National Clinical Research Center for Cancer/Cancer Hospital, Chinese Academy of Medical Sciences and Peking Union Medical College, Langfang, China; ^3^ Department of Nuclear Medicine, State Key Laboratory of Complex Severe and Rare Diseases, Peking Union Medical College Hospital Chinese Academy of Medical Sciences and Peking Union Medical College, Beijing, China; ^4^ Department of Nuclear Medicine, Weifang People’s Hospital, Weifang, Shandong, China

**Keywords:** esophageal neuroendocrine neoplasm, FDG, PET/CT, metabolic tumor volume, prognosis

## Abstract

**Objective:**

The aim of this bicentric retrospective study was to assess the prognostic value of ^18^F-FDG PET/CT in patients with esophageal high-grade neuroendocrine carcinoma (NECs).

**Methods:**

From the database of two centers, 28 patients affected by esophageal high-grade NECs who underwent ^18^F-FDG PET/CT before treatment were retrospectively reviewed. Metabolic parameters (SUVmax, SUVmean, tumor-to-blood-pool SUV ratio (TBR), tumor-to-liver SUV ratio (TLR), metabolic tumor volume (MTV), total lesion glycolysis (TLG)) of the primary tumor were measured. Univariate and multivariate analyses were performed for progression-free survival (PFS) and overall survival (OS).

**Results:**

After a median follow-up period of 22 months, disease progression occurred in 11 (39.3%) patients, and death occurred in 8 (28.6%) patients. The median PFS was 34 months, and the median OS was not reached. Univariate analyses revealed that among metabolic parameters, only MTV and TLG were significant prognostic factors, while among clinical variables, only distant metastasis was a significant factor for both PFS and OS (P< 0.05). On multivariate analyses, MTV and TLG were independent prognostic factors for both PFS and OS (P< 0.05).

**Conclusions:**

In patients with esophageal high-grade NEC, MTV and TLG measured on pretreatment ^18^F-FDG PET/CT are independently prognostic factors for predicting PFS and OS, and might be used as quantitative prognostic imaging biomarkers.

## Introduction

Esophageal cancer is an aggressive malignant disease and surgery remains the main potentially curative treatment. Squamous cell carcinoma (SCC) and adenocarcinoma (AC) are the two most common pathological types and account for over 95% of esophageal malignant diseases (1). Neuroendocrine carcinoma (NEC) arising from the esophagus is extremely rare, representing approximately 1% of esophageal cancer, and its incidence is reported to be higher in Asian countries than in Western countries (2). According to the 2022 WHO classification, neuroendocrine neoplasms (NENs) are divided into well-differentiated neuroendocrine tumors (NETs) and poorly differentiated neuroendocrine carcinomas (NECs). NETs are further subdivided into G1 (Ki-67 index: <3%), G2 (Ki-67 index: 3%~20%), and G3 (Ki-67 index: >20%) based on proliferation, whereas NECs (Ki-67 index: >20%) are by definition high grade (3). The WHO classified the neuroendocrine neoplasm (NEN) into grades 1, 2, and 3 according to mitotic count and proliferation index (4). NEC is defined as poorly differentiated carcinoma with a proliferation index >20% and/or mitotic count of >20 per10 high power field. NECs of the esophagus can be classified into small and large cell types. Esophageal NECs are associated with a worse survival rate compared to SCCs and ACs of the esophagus, and local or distant metastases often occurred at the time of diagnosis (1). There are no unique staging systems and treatment guidelines for esophageal NEC, and the treatment strategies in most centers follow that for lung and gastroenteropancreatic (GEP) NEC (4–8). Due to its rarity, only a few studies of esophageal NEC have been reported in the literature, with most focusing on the clinicopathological features and treatment (1, 9–14).

^18^F-FDG PET/CT is well established for the staging, restaging, and treatment response evaluation of various cancers. However, data on the use of ^18^F-FDG PET/CT in esophageal NEC is limited. By far, only a few case studies have specifically evaluated ^18^F-FDG uptake in esophageal NEC, which showed that lesions were hypermetabolic and ^18^F-FDG was helpful in staging and therapeutic management (15, 16). It is well known that ^18^F-FDG PET/CT is useful for evaluating NEN patients. Moreover, ^18^F-FDG uptake of the NEN lesions has been shown to provide prognostic information beyond that offered by WHO grading (17). However, these studies mostly included NENs of GEP origin and of all grades (grade 1–3). Different from GEP NENs, esophageal NEN mostly comprised of high-grade NECs, and are biologically more aggressive. The prognostic information provided by ^18^F-FDG PET/CT in GEP NENs may not be applicable to esophageal high-grade NECs. Nowadays, because of the rarity of the disease, no studies concerning the prognostic usefulness of ^18^F-FDG PET/CT in esophageal high-grade NEC are available. Herein, we conducted this retrospective study to investigate whether the metabolic parameters from pretreatment ^18^F-FDG PET/CT scan could be predictive of the outcome in patients with esophageal high-grade NECs.

## Methods

### Patients

We retrospectively reviewed the data of 112 patients with histopathologically confirmed esophageal high-grade NECs who underwent ^18^F-FDG PET/CT between September 2012 and January 2022 from 2 institutions (Cancer Hospital Chinese Academy of Medical Sciences, and Peking Union Medical College Hospital). Patients who had undergone a baseline pretreatment ^18^F-FDG PET/CT were enrolled in the study. The exclusion criteria were: (1) history of another malignant disease, (2) patients who had a history of anti-tumor treatment, (3) patients whose follow-up data were unavailable, (4) follow-up duration less than 6 months for patients who survived without disease progression, (5) patients whose deaths were caused by diseases other than esophageal NEC during the follow-up. Finally, 28 patients met the criteria and were included in this study. This retrospective study was approved by the institutional review board, and patient consents were not required due to the retrospective design.

### ^18^F-FDG PET/CT study

All PET/CT scans were performed with dedicated PET/CT scanners (Biograph 64 Truepoint True V, Siemens; Discovery 690, GE Healthcare) following the standard protocol. Before ^18^F-FDG injection, all patients fasted at least 4-6 hours to ensure that the blood glucose level was less than 120 mg/dL. The PET/CT scans were started approximately 60 minutes after the intravenous administration of ^18^F-FDG (5.5 MBq/kg). A low-dose CT scan from the upper thigh to the base of the skull was obtained for attenuation correction and anatomical localization. PET scans were acquired in a 3D mode for 2-3 minutes per bed position.

### Measurement of PET parameters

^18^F-FDG PET/CT images were retrospectively read by 2 experienced nuclear medicine physicians (GH, 7 years of experience; NZ, 10 years of experience). The values of SUVmax, SUVmean, metabolic tumor volume (MTV), and total lesion glycolysis (TLG) were measured at MIM workstation (version 6.6.11, MIM Software, USA). A rectangular semiautomatic volume of interest (VOI) was set to comprise the entire tumor. Tumor contours were first semiautomatically segmented with a SUV cutoff of 2.5. The tumor contours were then manually adjusted to avoid the physiological uptake (e.g., heart, urinary tract, brain, vocal cords, liver, spleen, brown fat tissue). Afterward, the software automatically measured the SUVmax, SUVmean, MTV, and TLG of the tumor. The tumor SUVmax to blood pool mean SUVratio (TBR), and the tumor SUVmax to liver mean SUVratio (TLR) were also obtained. To obtain the mean SUV for the blood pool and the liver, VOIs were drawn from the area distal to the aortic valve in the ascending aorta and the center of the right liver lobe, respectively.

### Statistical analysis

The prognostic value of metabolic parameters was investigated using progression-free survival (PFS) and overall survival (OS). PFS was defined as the time from initiation of treatment to the date of documented progression, while OS was measured from the date treatment to the date of death. Median follow-up was calculated using the reverse Kaplan-Meier method. For patients without disease progression, the time of the last follow-up was used as the endpoint. Receiver operating characteristic (ROC) curve analysis was performed to calculate the cut-off values of PET parameters for predicting survival. The area under the curve (AUC) was used to assess the accuracy of the prognostic factor. In univariate analysis, survival curves were estimated using the Kaplan-Meier method for each variable and were compared using the log-rank test to evaluate for any statistical significance. The Cox proportional hazards model was used in multivariate analysis with variables with *P* values of < 0.05 in univariate analysis to identify independent prognostic factors for PFS and OS. Correlations between PET parameters were examined using Spearman’s rank correlation coefficient (rho). Continuous data were expressed as mean ± standard deviation. P < 0.05 was considered statistically significant. All statistical analyses were performed using SPSS (IBM SPSS Statistics for Windows, Version 21.0. Armonk, NY).

## Results

### Patient characteristics

A total of 28 esophageal high-grade NEC patients were enrolled in this study and the baseline characteristics of patients are summarized in **Table 1**. There were 19 men and 9 women with a median age of 66 years (range, 46–87 years). At baseline, lymph node metastasis was observed in 17 patients, distant metastasis was observed in 4 patients. Stage data was available for 24 patients. According to the 8th edition of the American Joint Committee on Cancer (AJCC) staging system for esophageal squamous cell carcinoma, 7 patients were at stage I, 5 patients stage II, 6 patients stage III, 6 patients stage IV (18). Of the 28 patients, the histopathological type was small cell NEC in 12, large cell NEC in 8, and unclassified NEC in 8. Ki-67 proliferation index data were available in 25 patients and ranged from 60% to 95%, with a median value of 80%.

**Table 1:** Baseline characteristics of patients.

Characteristics	Value
Age, median (range) years	66 (46–87)
Gender, n (%)	
Male Female	19 (67.9%) 9 (32.1%)
Histopathology, n (%)	
Small cell neuroendocrine carcinoma Large cell neuroendocrine carcinoma Unclassified	12 (42.9%) 8 (28.6%) 8 (28.6%)
Ki-67, n (%)	
≤ 80% > 80%	12 (48%) 13 (52%)
T stage, n (%)	
T1 T2 T3 T4 Tx	6 (21.4%) 4 (14.3%) 5 (17.9%) 1 (3.6%) 12 (42.9)
N stage, n (%)	
N0 N1	11 (39.3%) 17 (60.7%)
M stage, n (%)	
M0 M1	24 (85.7%) 4 (14.3%)
Treatment	
Surgery Chemotherapy Radiotherapy Immunotherapy	16 (57.1%) 15 (53.6%) 12 (42.9%) 2 (7.1%)

^18^F-FDG PET/CT showed increased activity in all primary tumors with a wide range of SUVmax values (range, 3.1–28.4; median, 13.1). The values of the investigated metabolic parameters were summarized in **Table 2**. ^18^F-FDG PET/CT identified metastatic lesions in 9 patients, including lymph node metastases in 17, liver metastases in 4, lung metastases in 1, bone metastases in 1.

**Table 2:** Summary of investigated metabolic parameters.

Parameters	Mean ± SD	Range
SUVmax	14.3 ± 7.5	3.1–28.4
SUVmean	6.5 ± 3.0	2.25–13.85
TBR	8.7 ± 5.3	2.1–22.2
TLR	6.1 ± 3.4	1.5-13.3
MTV	31.0 ± 47.6	0.63–213
TLG	254.8 ± 428.9	2.08–1874.7

SUV, standardized uptake value; TBR, tumor-to-blood SUV ratio; TLR, tumor-to-liver SUV ratio; MTV, metabolic tumor volume; TLG, total lesion glycolysis.

### Prognostic factors for PFS and OS

After a median follow-up duration of 22 months (range, 6–96 months), disease progression occurred in 11 (39.3%) patients, and death occurred in 8 (28.6%) patients. The median PFS was 34 months, and the median OS was not reached. The estimated 1-year and 2-year PFS rates were 79.9% and 50.7%, respectively. The estimated 1-year and 2-year OS rates were 88.5% and 62.3%, respectively.

**Figure 1:**
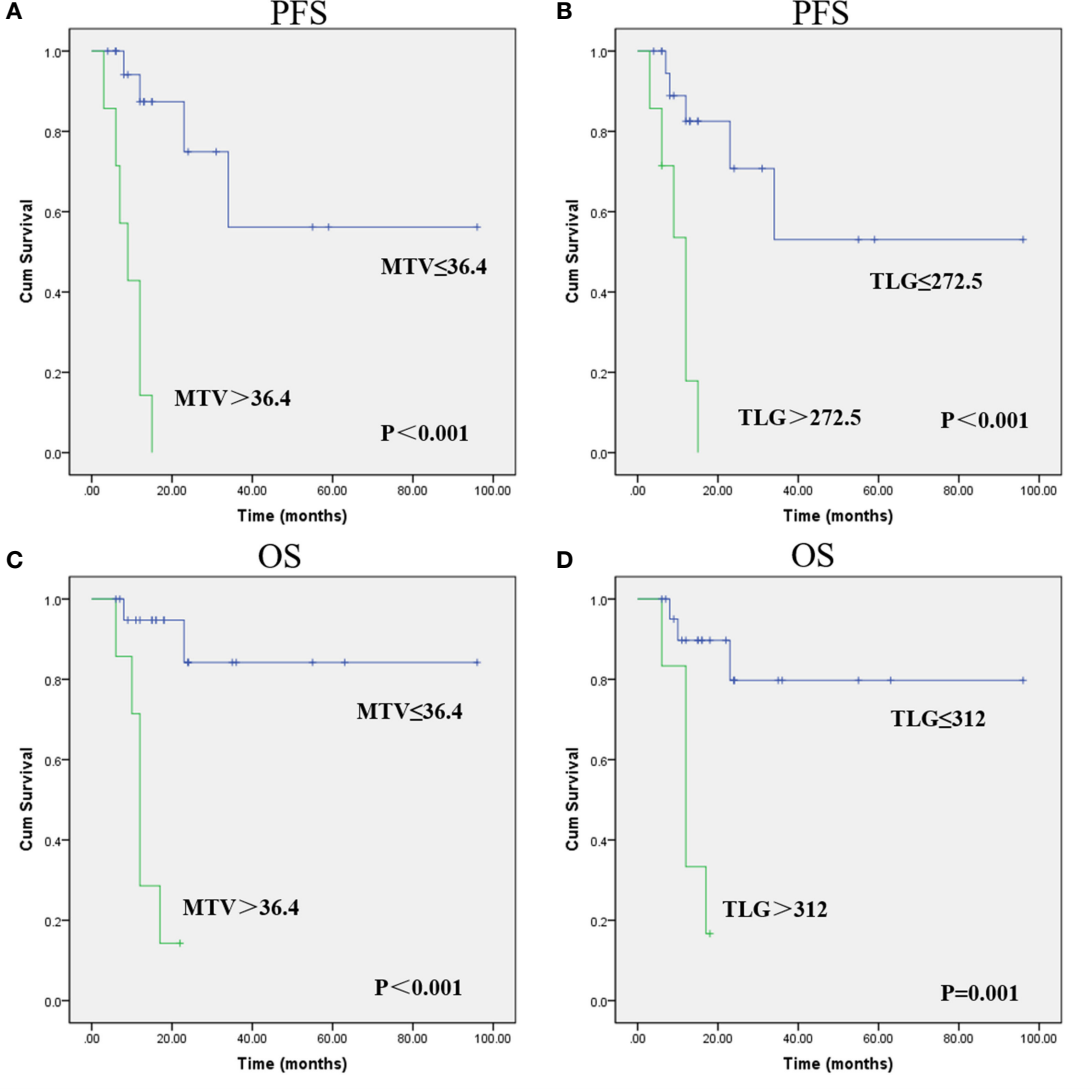
Progression-free survival (PFS) and overall survival (OS) curves were estimated using the Kaplan-Meier method. Kaplan-Meier survival graphs show significant differences in PFS **(A, B)** and OS **(C, D)** between the groups categorized according to MTV and TLG. (Cum Survival: Cumulative Survival).

**Figure 2:**
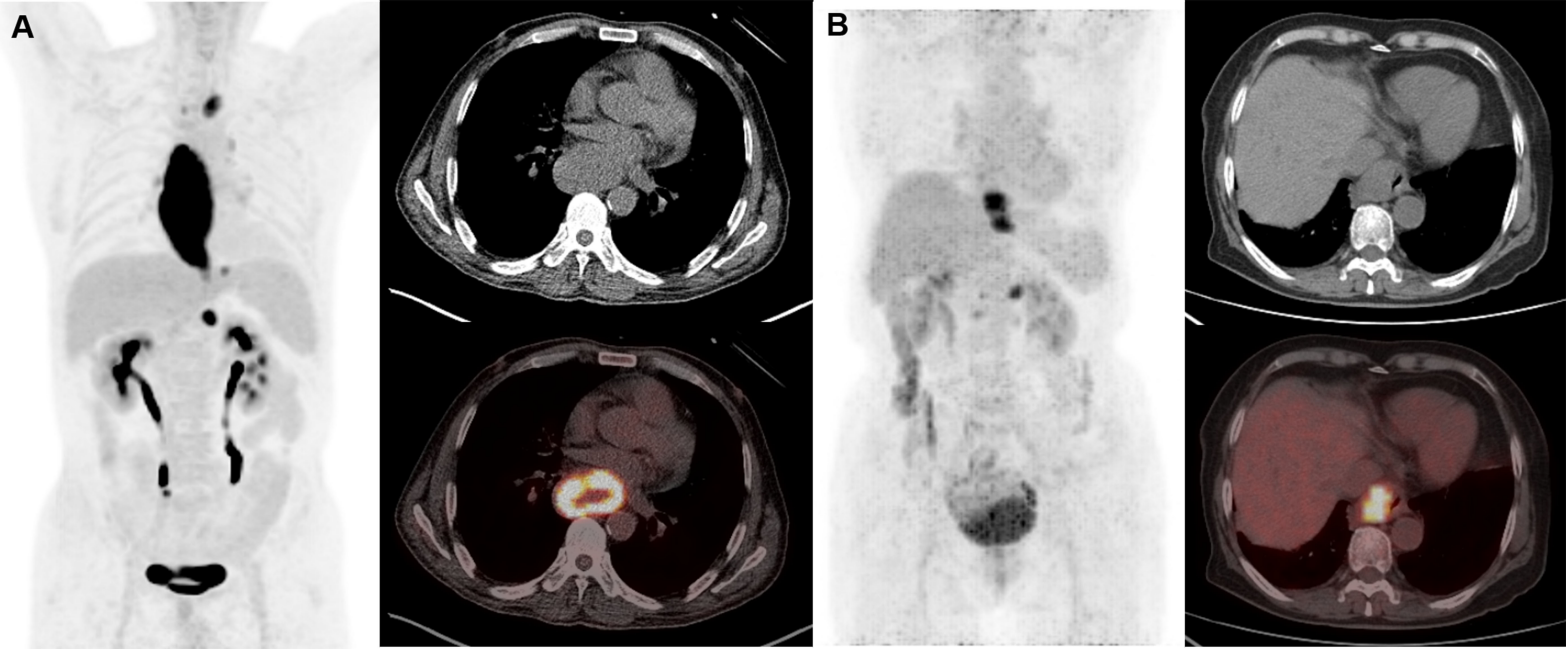
Representative cases of metabolic parameters affecting prognosis. Patient A was a 60-year-old man (SUVmax 23.9, SUVmean 8.8, TBR 16.0, TLR 11.3, MTV 213, TLG 1874.7). He had lymph node metastases at initial diagnosis and received chemotherapy. This patient suffered from lung recurrence and died from the disease at 12 months after initial treatment. Patient B is a 76-year-old woman (SUVmax 8.7, SUVmean 4.6, TBR 6.4, TLR 4.5, MTV 22.9, TLG 104.9). She had lymph node metastases at initial diagnosis and received radiotherapy and chemotherapy. This patient remains progression free for 96 months and is still alive until the last follow-up. Compliance with ethical standards.

The optimal cutoff points of PET parameters identified by ROC analysis are summarized in **Table 3**. Kaplan–Meier analysis revealed that patients with higher MTV (>36.4) and TLG (>272.5) had a significantly worse PFS than those with lower MTV (≤36.4; median PFS, 9 months *vs*. not reached; P<0.001; **Figure 1A**) and TLG (≤272.5; median PFS, 12 months *vs*. not reached; P<0.001; **Figure 1B**). Also, patients with higher MTV (>36.4) and TLG (>312.0) had a significantly poorer OS than those with lower MTV (≤36.4; median OS, 12 months *vs*. not reached; P<0.001; **Figure 1C**) and TLG (≤312.0; median OS, 12 months *vs* not reached, P = 0.001; **Figure 1D**). Representative images of ^18^F-FDG PET/CT quantification in 2 patients are shown in **Figure 2**.

**Table 3:** Metabolic PET/CT parameters cutoff calculated using receiver operating characteristic (ROC) curve analysis.

Parameter	ROC curve for PFS					ROC curve for OS				
	cutoff	AUC (95%CI)	P value	Sensitivity	Specificity	cutoff	AUC (95%CI)	P value	Sensitivity	Specificity
SUVmax	6.27	0.652 (0.448-0.857)	0.180	100%	35.3%	8.74	0.700 (0.487-0.913)	0.104	100%	40%
SUVmean	6.21	0.570 (0.356-0.783)	0.541	54.5%	64.7%	6.82	0.616 (0.388-0.843)	0.347	62.5%	70%
TBR	3.83	0.631 (0.423-0.839)	0.249	100%	33.3%	4.61	0.656 (0.441-0.871)	0.204	100%	40%
TLR	2.61	0.636 (0.428-0.845)	0.230	100%	35.3%	2.91	0.663 (0.439-0.886)	0.186	100%	35%
MTV	36.4	0.824 (0.658-0.989)	0.004	63.6%	100%	36.4	0.863 (0.699-1.000)	0.003	75%	95%
TLG	272.5	0.749 (0.554-0.943)	0.029	54.5%	94.1%	312.0	0.806 (0.605-1.000)	0.013	62.5%	95%

PFS, progression-free survival; OS, overall survival; AUC, area under curve; CI, confidence interval; SUV, standardized uptake value; TBR, tumor-to-blood SUV ratio; TLR, tumor-to-liver SUV ratio; MTV, metabolic tumor volume; TLG, total lesion glycolysis.

The result of univariate analysis is shown in **Table 4**. The univariate analysis revealed that among clinical factors, only distant metastasis was associated to PFS and OS (P<0.05). Among PET parameters, SUVmax, SUVmean, TBR, TLR were not related to outcome survival, both for PFS and OS. Instead, MTV and TLG were significantly correlated with PFS and OS. As MTV and TLG were significantly correlated (*r*=0.964, P<0.001), we used two different models (including MTV and TLG separately) to perform multivariate analysis. The multivariate analysis revealed that both MTV and TLG were independent prognostic factor for PFS and OS (P<0.05). **Table 5** demonstrates the result of multivariate analysis.

**Table 4:** Univariate analysis for progression-free survival (PFS) and overall survival (OS).

Variables	PFS		OS	
	P value	HR (95%CI)	P value	HR (95%CI)
Gender	0.953	0.963 (0.280-3.312)	0.978	1.021 (0.239-4.366)
Age	0.143	0.368 (0.096-1.404)	0.613	0.691 (0.165-2.894)
Histologic type	0.835	1.159 (0.288-4.675)	0.479	1.813 (0.350-9.402)
Ki-67	0.212	2.527 (0.589-10.841)	0.563	1.695 (0.283-10.149)
Lymph node metastasis	0.062	0.221 (0.045-1.081)	0.170	0.018 (0.000-5.583)
Distant metastasis	**0.013**	0.186 (0.050-0.697)	**0.046**	0.230 (0.055-0.973)
Surgery	0.276	2.128 (0.548-8.273)	0.147	3.277 (0.660-16.277)
Chemotherapy	0.103	0.277 (0.059-1.294)	0.119	0.188 (0.023-1.541)
SUVmax	0.297	0.033 (0.000-19.968)	0.267	0.027 (0.000-15.767)
SUVmean	0.452	0.632 (0.191-2.088)	0.279	0.452 (0.107-1.903)
TBR	0.297	0.033 (0.000-19.968)	0.289	0.029 (0.000-20.070)
TLR	0.297	0.033 (0.000-19.968)	0.322	0.031 (0.000-29.459)
MTV	**0.001**	0.065 (0.013-0.317)	**0.005**	0.049 (0.006-0.408)
TLG	**0.002**	0.112 (0.027-0.452)	**0.008**	0.107 (0.021-0.557)

SUV, standardized uptake value; TBR, tumor-to-blood SUV ratio; TLR, tumor-to-liver SUV ratio; MTV, metabolic tumor volume; TLG, total lesion glycolysis. Bold represents values less than 0.05.

**Table 5:** Multivariate analysis for progression-free survival (PFS) and overall survival (OS).

	Variables	Model A*		Model B*	
		P value	HR (95%CI)	P value	HR (95%CI)
PFS	Distant metastasis	0.343	0.500 (0.119-2.099)	0.136	0.342 (0.084-1.399)
	MTV	**0.004**	0.084 (0.016-0.451)	–	–
	TLG	–	–	**0.012**	0.153 (0.036-0.660)
OS	Distant metastasis	0.367	0.500 (0.111-2.255)	0.168	0.352 (0.080-1.551)
	MTV	**0.011**	0.059 (0.007-0.515)	–	–
	TLG	–	–	**0.017**	0.131 (0.024-0.698)

*Due to the multicollinearity between MTV and TLG, two models, including MTV and TLG separately, were performed. HR, hazard ratio; CI, confidence interval; MTV, metabolic tumor volume; TLG, total lesion glycolysis. Bold represents values less than 0.05.

## Discussion

Esophageal high-grade NEC is a rare malignant tumor of the esophagus, but despite the relatively low prevalence, the incidence rate is steadily increasing (6). Esophageal NEC is radically different from SCC and AC in biological behavior and prognosis. Clinically, Ki-67 index can be considered as an indicator of tumor cell proliferation. The fact that the patients in the present study had a median Ki-67 value of 80% also indicates the high malignancy of this entity. In this study, we investigated the prognostic usefulness of metabolic parameters (SUVmax, SUVmean, TBR, TLR, MTV, and TLG) derived from pretreatment ^18^F-FDG PET/CT in patients with esophageal high-grade NEC. Our results demonstrated that, among metabolic parameters, only MTV and TLG of primary tumor were significant predictors for both PFS and OS in univariate analysis, and remained independent even after adjusting for known prognostic factor (distant metastasis) in multivariate analysis.

NETs are a heterogeneous group of neoplasms with a diverse clinical outcome. Most studies applying ^18^F-FDG PET/CT in NEN include all tumor grades. As far as we know, only a few studies have looked at the prognostic value of ^18^F-FDG PET/CT in high-grade NEN. Jiang et al. reported the prognostic value of ^18^F-FDG PET/CT in 22 patients with cervical NEC (19). Similar to our study, they found that, MTV and TLG, instead of SUVmax, were significant predictors of survival. Furthermore, Lim et al. investigated 27 patients with gastric NEC and mixed adenoneuroendocrine NEC (MANEC). Like our study, they found that patients with high MTV/TLG showed poorer prognosis compared to low MTV/TLG patients. In contrast, SUVmax did not predict survival outcome (20). In a recent study, Stokmo et al. evaluated 66 patients with high-grade GEP NEN, and found MTV and TLG were also stronger prognostic parameters than SUVmax (21). Several studies also reported the prognostic value of SUVmax in NEN with higher SUVmax predicting poor outcome (22–24). Different from our study, these studies included NEN of all grades, and it is expected that high-grade NENs, which have high Ki-67 index (> 20%) and poor prognosis, demonstrate higher SUVmax than low-grade NENs. Besides, SUVmax is determined by a single highest voxel activity within a tumor and may not reflect the whole metabolic and volumetric burden of the tumor (25). These findings support the superiority of MTV and TLG over SUVmax for predicting survival outcome in esophageal NEC.

In a recent study by Chen et al., which included 283 patients with esophageal NEC and over half (53.4%) of the patients presented with distant metastasis at the time of diagnosis, distant metastasis was found to be significantly related to worse survival outcome (26). This is partly consistent with our finding. Our results considered that distant metastasis was significantly associated with worse PFS and OS in univariate analysis, while failed to remain an independent risk factor in multivariate analysis, which might be due to the small number of patients in this study. Nevertheless, after adjusting for distant metastasis, MTV and TLG still remain independent in multivariate analysis. The fact that distant metastasis is a known predictor of outcome further highlights the prognostic potential of MTV and TLG. In addition, lymph node metastasis (27, 28), Ki-67 index (13), surgery (26), chemotherapy (26) have also been suggested as significant predictors of survival in esophageal NEC in previous studies. However, the univariate analysis in our study did not reveal significant association between these clinical factors and survival.

This study has several limitations. First, the retrospective design of the study might impose potential selection bias, which might be mitigated, at least partly, by combining the patients from the 2 institutions. The second limitation was that the number of the patients involved in this study is small, and the patient number in subgroups varies significantly, which might impact the resulting accuracy. Third, the follow-up duration is not long enough, death occurred only in around one third of patients, limiting the statistical power to robustly evaluate the prognostic implications of clinical and imaging factors on OS. In addition, the use of different treatment procedures among patients due to uncertainty in management guidelines is also another limitation, which might exert some impact on the clinical outcome. Fourth, somatostatin receptor (SSTR)-targeted PET/CT is a valuable imaging method for well-differentiated NEN. However, due to the retrospective design of the present study, we could only use the existing data. No patients with esophageal NECs underwent SSTR PET/CT, thus the comparison between SSTR PET/CT and FDG PET/CT could not be performed. Lastly, quantitative parameters indicating FDG metabolism might be influenced by vendor specific characteristics, which differ between different scanners. This might impact the resulting accuracy.

## Conclusions

In patients with esophageal high-grade NEC, MTV and TLG measured on pretreatment ^18^F-FDG PET/CT are independently associated with PFS and OS, and might be used as quantitative prognostic imaging biomarkers.

## Data availability statement

The original contributions presented in the study are included in the article/supplementary material. Further inquiries can be directed to the corresponding authors.

## Ethics statement

This retrospective study was approved by the institutional review board of National Cancer Center/National Clinical Research Center for Cancer/Cancer Hospital, Chinese Academy of Medical Sciences and Peking Union Medical College and Peking Union Medical College Hospital. The ethics committee waived the requirement of written informed consent for participation.

## Author contributions

GH, NZ, FL, HJ, and RZ contributed to the design and implementation of the research, to the analysis of the results, and to the writing of the manuscript. All authors contributed to the article and approved the submitted version.
